# Transparent Pneumatic Tactile Sensors for Soft Biomedical Robotics

**DOI:** 10.3390/s23125671

**Published:** 2023-06-17

**Authors:** Sinuo Zhao, Chi Cong Nguyen, Trung Thien Hoang, Thanh Nho Do, Hoang-Phuong Phan

**Affiliations:** 1 School of Mechanical and Manufacturing Engineering, UNSW Sydney, Kensington Campus, Sydney, NSW 2052, Australia; sinuo.zhao@student.unsw.edu.au; 2 Tyree Institute of Health Engineering, UNSW Sydney, Sydney, NSW 2052, Australia; cong.c.nguyen@unsw.edu.au (C.C.N.); trungthien.hoang@unsw.edu.au (T.T.H.); 3 Graduate School of Biomedical Engineering, Faculty of Engineering, UNSW Sydney, Kensington Campus, Sydney, NSW 2052, Australia; tn.do@unsw.edu.au

**Keywords:** transparent pressure sensors, robotic palpation, surgical soft robotics, flexible sensors

## Abstract

Palpation is a simple but effective method to distinguish tumors from healthy tissues. The development of miniaturized tactile sensors embedded on endoscopic or robotic devices is key to achieving precise palpation diagnosis and subsequent timely treatment. This paper reports on the fabrication and characterization of a novel tactile sensor with mechanical flexibility and optical transparency that can be easily mounted on soft surgical endoscopes and robotics. By utilizing the pneumatic sensing mechanism, the sensor offers a high sensitivity of 1.25 mbar and negligible hysteresis, enabling the detection of phantom tissues with different stiffnesses ranging from 0 to 2.5 MPa. Our configuration, combining pneumatic sensing and hydraulic actuating, also eliminates electrical wiring from the functional elements located at the robot end-effector, thereby enhancing the system safety. The optical transparency path in the sensors together with its mechanical sensing capability open interesting possibilities in the early detection of solid tumor as well as in the development of all-in-one soft surgical robots that can perform visual/mechanical feedback and optical therapy.

## 1. Introduction

Cancer is the leading cause of death worldwide, with nearly 10 million people dying from cancer in 2020, accounting for almost one-sixth of the total global deaths [1]. Early-stage and precise detection of tumor tissues is critically important, as timely cancer treatment can effectively reduce mortality rates [1]. Recent studies have suggested that the solid tumor progression typically increases collagen deposition and cross-linking within tissue stroma that deregulates and disorganizes the extracellular matrix (i.e., ECM, a three-dimensional assembly of macromolecules and interconnected cell-scale fibres) [2]. Such changes in the ECM result in a modification in the biochemical and mechanical properties of cells, where many solid tumors are significantly stiffer than normal tissues. This phenomenon makes stiffness-based inspection (i.e., palpation) a relatively accurate but simple method to detect cancerous tissues, through which medical doctors can use their sense of touch to determine tumors [3,4,5,6]. However, touch sensing in humans is subjective; therefore, objective methods that employ digital sensors integrated into endoscopes or medical robotics represent attractive features for tumor detection combined with robot-assisted treatment. The implementation of soft robotic palpation is expected to enhance diagnostic efficiency and accuracy, and, at the same time, reduce tissue damage through minimally invasive procedures.

There are several types of tissue palpation devices with different structural configurations and sensing mechanisms. Among these, force [7,8,9] and pressure [10,11,12,13] sensors have been widely used to correlate the induced mechanical stimuli from the robot with the deformation of tissues to quantify tissue stiffness. These sensors are designed through different operating mechanisms, for instance, devices based on indentation depth use large and smooth contact surfaces to accurately measure tissue characteristics [11,14]. Sharp probe-type instruments offer higher-resolution tissue stiffness features but can cause tissue damage due to their small contact area [7,15]. Arrays of tactile sensors with pneumatic devices for sensing provide higher spatial resolution than single-point force feedback, and reduce tissue damage during contact [16,17]. In addition, tactile sensors that mimic the human finger structure can increase the accuracy of detecting hard nodules in soft tissue [18,19]. Furthermore, combining tactile sensing with wireless communication can eliminate the need for complicated wiring and electrical interconnect that can enhance robotic manoeuvre capability and reduce the incision size [10,20].

Enhancing precision and sensitivity has been an area of focus in the development of tactile sensors. Common working mechanisms utilized in tactile sensors can be categorized as follows: piezoresistive effect, capacitive effect, piezoelectric effect, triboelectric effect, and optical-based effect [21]. Table 1 lists some representative examples of sensors that were developed based on these sensing effects. These sensors demonstrate performance improvements through intricate fabrication processes and the utilization of diverse materials.

Despite the advances in tactile sensors, most available sensors were developed for dexterous robotic hands. The development of tactile sensors integrated into endoscope and soft robotics remains challenging due to (i) the difficulty to install sensors on a small footprint, (ii) the requirement for mechanical flexibility to retain robotic manoeuvrability within a small workspace inside the human body, and (iii) the need for optical transparency to allow real-time observation and assessment. In recent research, scientists have been devoted to developing tactile sensors with higher precision and lower dimensions. Wang et al. [27] designed a high-quality tactile matrix sensor array based on the field-effect transistor principle, aiming to achieve a highly integrated, scalable, and multifunctional FET tactile sensor with small size and high sensitivity. Similarly, a MEMS-based force sensor [28] was integrated into a manipulable robotic probe for precise detection of tumor tissue edges during excision procedures. However, this type of sensor faces challenges in mitigating temperature effects on sensors and lacks the ability to capture visual information due to the absence of optical transparency. There have also been attempts to incorporate visual information into tactile sensors. Cho et al. [29] designed a miniature probe that utilizes light-scattering principles for tactile image analysis to directly diagnose thyroid cancer. Kara et al. [30] developed a visual-based surface tactile sensor (VS-TS) to extract morphological features (i.e., shape, texture) and stiffness characteristics of CRC polyps for the early diagnosis of colorectal cancer (CRC). However, these sensors are relatively large and still only acquire blurred visual images. The summarized details of the above studies are presented in Table 2.

To overcome the above bottlenecks, this work develops a novel soft, pneumatic tactile sensor for minimally invasive soft robotics. The experimental results demonstrate that the sensor can detect phantom tissue models with stiffnesses ranging from 0 to 2.5 MPa. The mechanical flexibility of the sensors allows for a simple integration into soft robots, offering a high degree of freedom with different motions such as bending and linear actuations, suitable for hard-to-reach organs. The optical transparency of the constructing material (PDMS) enables real-time observation which provides useful visual and mechanical feedback for tumor detection and localization.

## 2. Sensor Principle and Design

**Figure 1:**
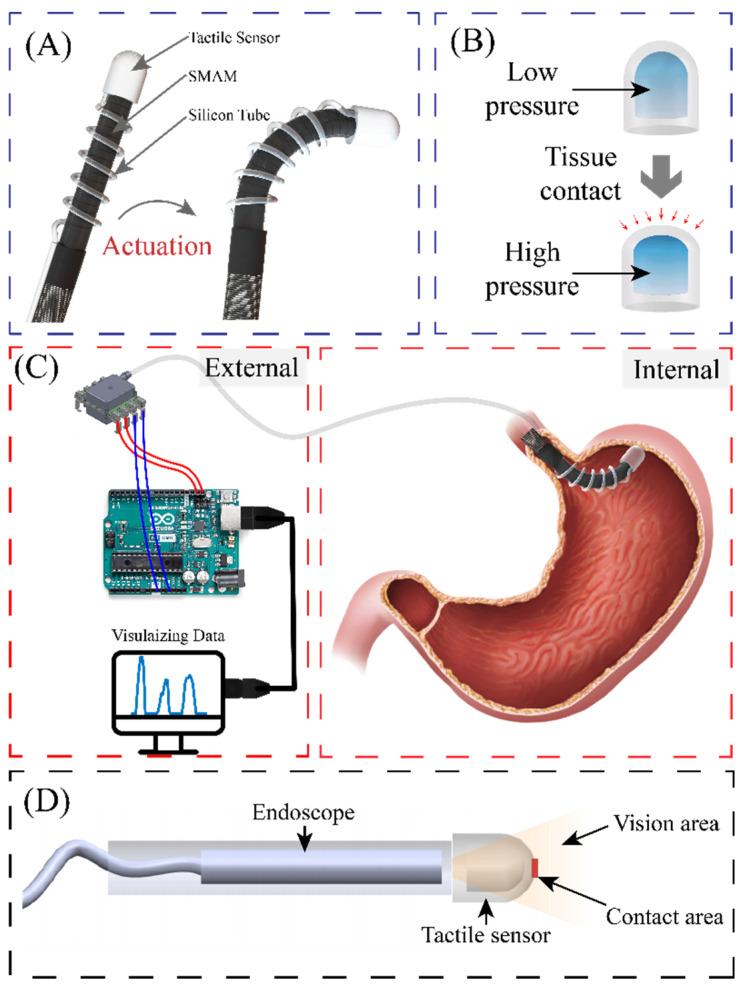
Schematic sketch of transparent pressure sensor integrated into soft robotic. (**A**) Configuration of soft robotic system integrated with tactile sensor. (**B**) Schematic diagram of air chamber. (**C**) Configuration of the electrical and mechanical components. (**D**) Visual feedback of the tactile sensor.

Figure 1A illustrates the configuration of a soft robotic system actuated by three hydraulic soft microtubule artificial muscles (SMAM) and integrated with the proposed pneumatic tactile sensor. The tactile sensor is constructed by a transparent, dome-like air chamber and a commercial pressure-sensing element (Amphenol ELVH-M500G-HRND-C-N2A4) connected to the chamber via a silicon tube, and the silicon tube is wrapped around the soft robotic arm forming a spiral shape without affecting its bending motions. Unlike other tactile sensors, the end-effector (dome-like air chamber) of the proposed sensor with PDMS material has good elasticity, which is shown in Figure 1B, and it is installed in a soft surgical robot, thereby minimizing tissue damage caused by contact and retaining the mechanical flexibility of the system. This configuration (pneumatic sensing and hydraulic actuation) allows all electrical components (electric pump, control unit, and the pressure-sensing element) to be placed outside of the patient’s body, while only the mechanical parts (the hydraulic tube and the air chamber) are inserted through a small incision or through the gastrointestinal tract to reach the targeted organs, as depicted in Figure 1C. This design effectively eliminates the influence of temperature and enhances the safety of the soft robot by minimizing the electrical contact between functioning electronics with the surrounding tissues. Moreover, the proposed platform can address several technical limitations in the existing tactile sensing used in surgical robots. For instance, the soft tactile sensor reported by Campisano, F et al., [31] has the sensing component separated from the endoscope, which results in four incisions, potentially enlarging the patient’s wounds. Furthermore, the haptic sensor reported in [32] obstructs a portion of the captured images, limiting the overall field of view that can be obtained by the endoscope. Additionally, across all other design studies, the captured image perspective does not perfectly align with the tactile sensor’s position. As depicted in Figure 1D, the proposed sensor can offer a wide range of visual feedback perspectives with good image alignment.

The tactile sensor operates based on the change in the pressure level inside the air chamber when the robot contacts tissues. In particular, when the sensor is in contact with tissue, it deforms the PDMS dome that changes the volume of the air chamber, leading to a change in the pressure level (P_1_/P_2_ = V_2_/V_1_, where P_1_ and V_1_ are the pressure and volume before contact and P_2_ and V_2_ are the pressure and volume after contact, respectively). It should be noted that the silicone tube is not subject to direct external compression as it does not contact with tissue. Therefore, the deformation of the silicone tube is not taken into account when calculating the pressure equilibrium. Assuming that *h* is the displacement of the PDMS air chamber and the volume change in the entire deformation process can be represented by a polynomial containing the height, which can be approximated as the volume of a hemisphere with r as radius and h as height as follows:

(1) $$\begin{array}{ccc} V & = & \pi \times h^{2}\times (3r-h)/3\\ & = & -\frac{1}{3}\pi h^{3}+\pi rh^{2},h\in (0,r) \end{array}$$

According to this equation, the volume change in the entire deformation process can be expressed as a polynomial function of height. It is also assume that the volume change is proportional to the magnitude of the pressure. Therefore, the change in the pressure level is expected to be a polynomial function of the applied displacement. The mechanical stiffness of tissues can be distinguished by analysing the measured pressure and displacement.

**Figure 2:**
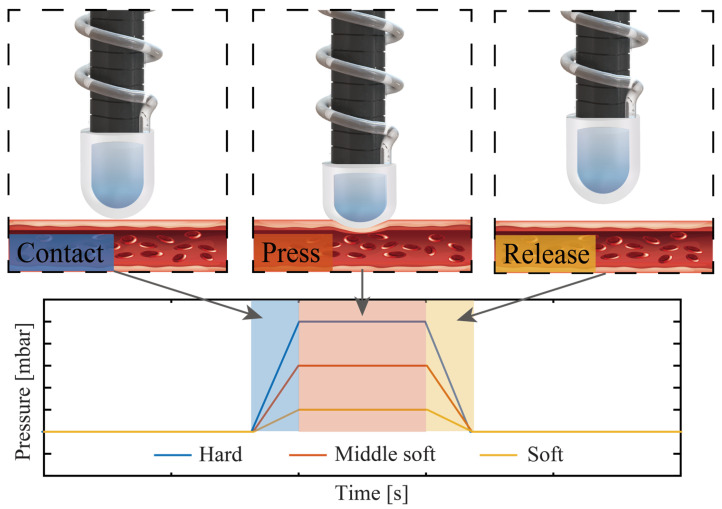
Demonstration of the tactile sensor for tumor detection.

Figure 2 illustrate the operating principle for robotic palpation using the proposed tactile sensors. Initially, the sensor is activated by the hydraulic SMAM to navigate to the designated area. Utilising the mechanical flexibility of the soft robot, it can manoeuvre and flex to uniformly exert pressure across various locations. The mechanical property of the tissue is evaluated by analysing the differential output data obtained from the pressure sensor.

## 3. Sensor Development

### 3.1. Mechanical Part

**Figure 3:**
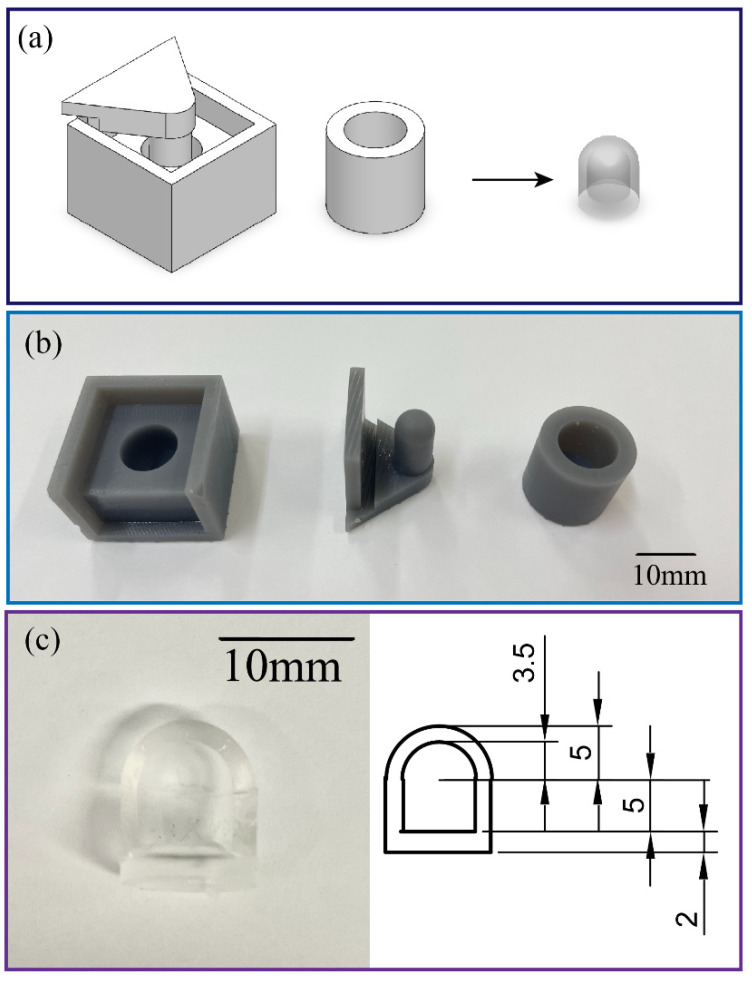
Fabrication process of the sensor. (**a**) A 3D model for 3D-printed mould and PDMS part. (**b**) Photograph of the 3D-printed moulds. (**c**) A photograph of the casted PDMS air chamber.

The air chamber (with a diameter of 10 mm, a height of 10 mm, and a wall thickness of 1.5 mm) of the tactile sensor was fabricated by casting PDMS (SYLGARD™ 184 Silicone Elastomer Kit with a mixing ratio of 10:1) into an SLA 3D-printed mould. The mould consists of two parts, the housing and the upper cap, which can be easily separated to detach the PDMS chamber from the mould. After vacuuming the PDMS dome-like structure to release air bubbles and curing at 60 °C for 2 h, the air chamber was sealed by bonding the PDMS dome with another layer of 2 mm thick PDMS film. An inlet was then punched out of the bottom of the chamber to insert the silicone tube (with an inner diameter of 0.8 mm, and an outer diameter of 1.6 mm) and connected to the commercial pressure sensor, as shown in Figure 3. The PDMS parts are elastic and hollow, which minimizes tissue damage when the robot interacts with targeted organs. The mechanical flexibility of the hollow structure combined with the low Young’s modulus of PDMS enables a large deformation of the chamber that results in a significant change in the inner pressure. The intrinsic optical transparency of PDMS avoids the issue related to the field-of-view blockage in traditional silicon or metallic-based sensors. This feature allows for real-time observation and visual feedback for the soft robot. The small footprint and electrical-wiring-free configuration are key features to ease the installation of the as-fabricated tactile sensor onto the tip of the soft robot.

Details of the fabrication process of the soft robotic arm can be found in our previous work [33]. It is noted that the integration of pneumatic soft sensors to the robotic arm retains the mechanical flexibility of the system as well as small incision size. Specifically, the tactile sensor was fixed to the soft robotics’ tip while the silicone tube was wrapped around the robot body using the Ecoflex 00-30 material as an adhesive with the curing process completed after two hours at 60 °C.

### 3.2. Readout Circuit

**Figure 4:**
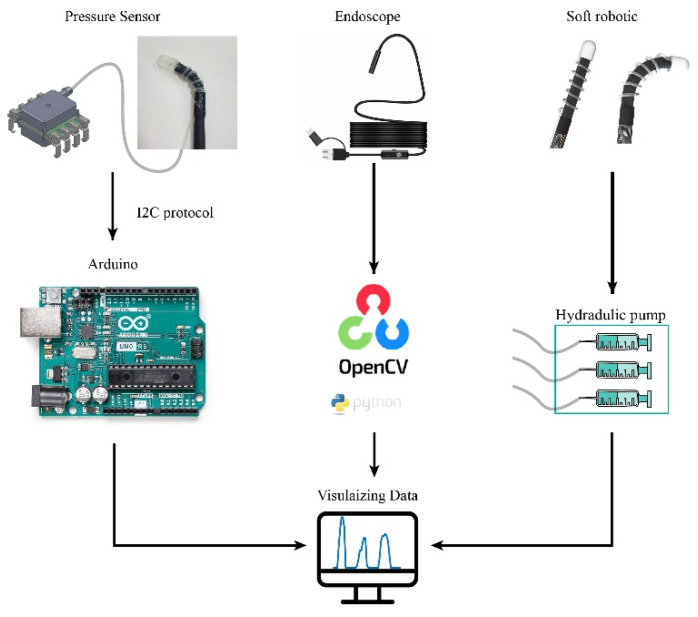
Configuration of the system with tactile sensors, optical path, and hydraulic actuator.

To obtain information of tissue stiffness, one side of the silicon tube was connected to the air chamber, while the other side was plugged into a commercial pressure sensor. The pressure sensor was connected to a controller Arduino^TM^ Uno Rev3 (Arduino LLC, Somerville, MA, USA) that records and transmits the measured data to a computer. The applied pressure (P) can be derived from the measured digital signal using the relationship: P=(((DigitalSignal−1638)/13,108)×500). Additionally, an optical fiber was placed underneath the air chamber to simultaneously observes and visualizes the inner tissues. The acquired images were stitched together using OpenCV (Open Source Computer Vision Library, vision 3.4.2) to obtain a larger field of view. The tactile sensor was manipulated by the soft robotic arm to obtain tissue stiffness at different positions. Finally, we implemented automatic data acquisition from both the sensor and endoscope using Python programming. The equipment connection layout and physical illustration are shown in Figure 4.

## 4. Results and Discussion

**Figure 5:**
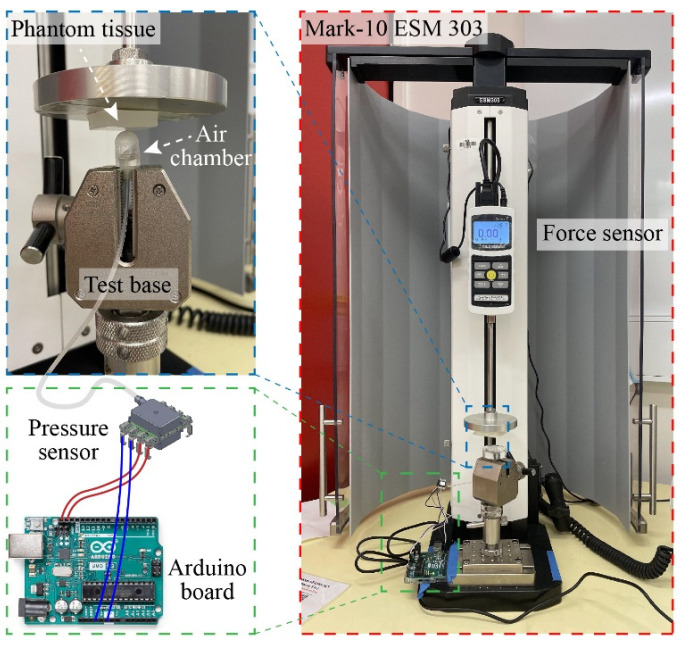
Photograph and schematic sketch of the experimental setup to calibrate the sensor.

Figure 5 shows the experimental setup for tactile sensor characterization. We utilized a motorized tension compression setup- Mark-10 ESM 303 (Mark-10 Corporation, Copiague, NY, USA), which is capable of real-time monitoring of the deformation (height) and applied force using Force Gauge Model M7-20 (Mark-10 Corporation, Copiague, NY, USA). The tactile sensor was firmly fixed onto the lower base of the compression setup while phantom tissues with different stiffnesses were mounted on the upper metallic stage (iron) and pressed against the sensor. In the calibration experiment, a flat metallic surface was pressed directly against the tactile sensors ensuring that all displacements are contributed by the deformation of elastic air chamber of the sensor. The sensor response (i.e., output pressure) and applied displacement was simultaneously measured, displayed, and recorded using an Arduino board.

**Figure 6:**
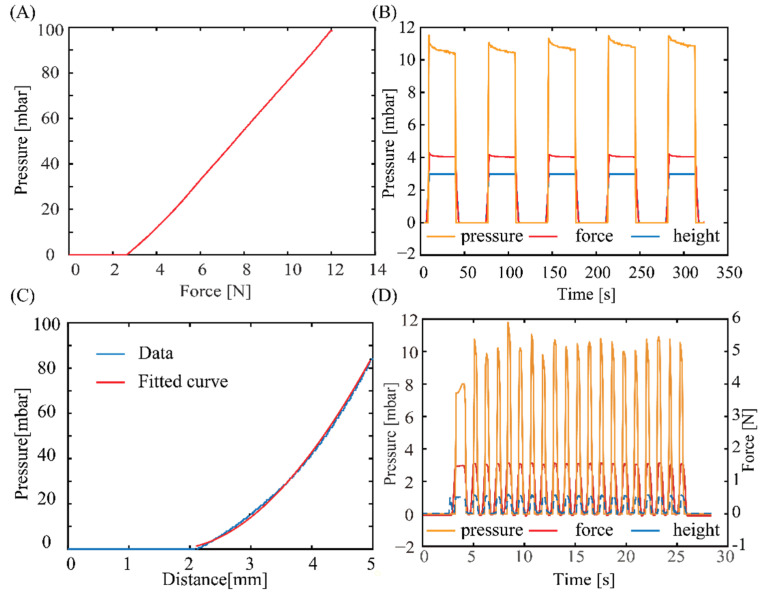
Response of the tactile sensors when pressed against a rigid substrate (iron). (**A**) Measured pressure vs. applied force. (**B**) The repeatability of the sensor after several pressurizing cycles (there are three variables with the same vertical axis, force measured in Newtons, and distance measured in millimetres). (**C**) The relationship between output pressure and applied deformation. (**D**) Dynamic response testing.

Figure 6A shows the response of the pressure sensor as the applied force varied. The response of the sensors started to be observable at an applied force of 2 N. The sensor output linearly increases with increasing the applied force from 2 N to 10 N, a typical range used for palpation application. From this relationship, the applied force can be extrapolated from the measured pressure: $F\left(N\right)\approx 0.095\times P+2.63$, which can serve as a feedback signal for robotic manipulation and palpation applications. Figure 6B shows the output of the sensors at a constant applied force and displacement. Evidently, the tactile sensor exhibits a good repeatability and stability after several pressurized cycles, indicating that air is well-sealed within the elastic chamber, the silicone tube, and the commercial pressure sensor. The tactile sensor does not show significant hysteresis once the applied pressure is released, indicating that PDMS air chamber can fully return to its initial shape owing to its excellent elasticity. Figure 6C plots the relationship between the measured pressure and applied displacement. The geometric deformation, based on Equation (1), can be fitted to the polynomial function: $P=-\frac{0.2831}{3}\pi \times (h-1.715{)}^{3}+0.2831\pi \times (h-1.715{)}^{2}\times 10$. The good agreement between the fitting curve and data provides evidence for the validity of the relationship in Equation (1). To assess the sensor’s capability to rapidly respond to applied forces at a high rate, we conducted a dynamic experiment by performing a 3 mm compression and lifting operation at a speed of 1100 mm/min. This compression cycle was repeated 20 times using the Mark-10 ESM 303 setup. As shown in Figure 6D, we can adjust the sampling frequency of the sensor to 10 Hz through the Arduino board to accurately capture pressure changes during rapid compression. Furthermore, the measured pressures were consistent over 20 cycles. 

Based on the stable performance of the tactile sensor in measuring pressure against hard substrates, we further demonstrated its capability in differentiating soft materials with different stiffnesses. Three phantom tissue models were prepared by using Ecoflex™ materials (Smooth-On, Inc., Macungie, PA, USA) with different elastic properties. The Young’s modulus of these phantom tissue can be estimated from the relationship between Shore and ISO hardness (s) and Young’s modulus (E) [34]: $E(MPa)=0.0981\left(56+7.66s\right)/0.137505\left(254-2.54s\right)$, where s is the Shore hardness. Accordingly, the approximate Young’s modulus values of Ecoflex™ 00-10, 00-30, and 00-50 are 0.41 MPa, 1.15 MPa, and 2.47 MPa, respectively.

**Figure 7:**
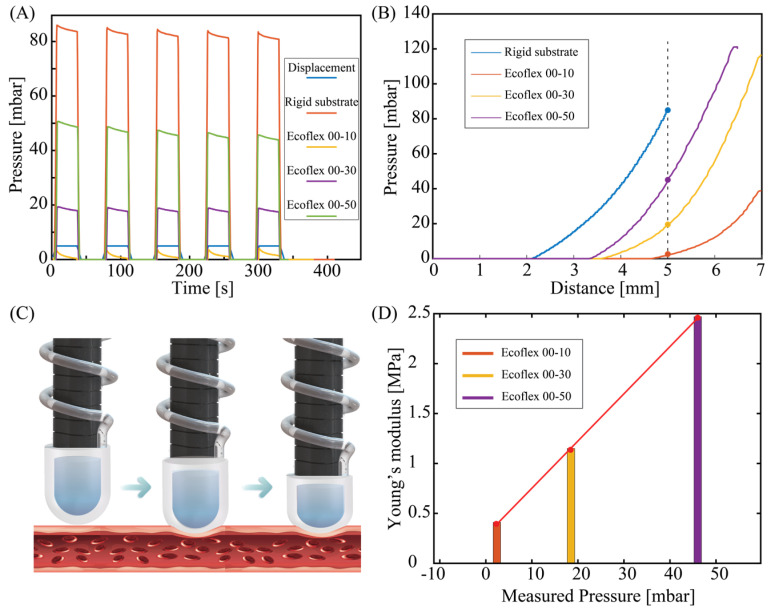
The output of sensors when pressing against tissue phantoms with different stiffnesses. (**A**) For all samples, the sensor exhibits good repeatability. (**B**) Transient response of the sensor when increasing pressurizing displacement. (**C**) Illustrates 2 stages of pressing the tactile sensor linearly onto the model tissue. (**D**) The relationship between measured pressure and the estimated Young’s modulus of each phantom tissue sample.

Figure 7A plots the response of the tactile sensor when the same 5 mm displacement is applied to all three phantom tissues and the rigid substrate (iron). All the cyclic pressurizing tests show a consistent sensor response with an output pressure of approximately 83.5 MPa measured for the rigid substrate (iron), while these values for Ecoflex 00-50, Ecoflex 00-30, and Ecoflex 00-10 are 46.03 Mpa, 18.5 Mpa, and 2.4 Mpa, respectively. Obviously, a higher pressure level is observed in the high stiffness phantom tissue (i.e., Ecoflex 00-50). To further interpret the relationship between the measured pressure output and the stiffness of the phantom tissue, we observed the transient response of the sensor when the applied displacement was linearly increased, as shown in Figure 7B. For all three types of phantom tissues, the tactile sensor exhibits a similar trend that the change in pressure is only observable when the displacement reached a certain threshold. Specifically, for the Ecoflex 00-50 sample, pressure change is observable at a displacement of around 3.4 mm, while this value for Ecoflex 00-30 and Ecoflex 00-10 is 3.7 mm and 4.7 mm, respectively.

The experimental data plotted in Figure 6A,C show a distinctive hysteresis characteristic in the proposed sensor. Specifically, the pressure reading remains at zero even after the initial pressing force is detected (by the Mark-10 ESM 303 setup) during the pressing process. This hysteresis becomes more pronounced when the phantom tissue is soft, as shown in Figure 7B. Figure 7C illustrates our hypothesis for this hysteresis phenomenon. During the initial compression phase, the tissue undergoes a deformation in a short time that does not affect the volume of the air chamber. In addition, as the tissue becomes softer, it prolongs the deformation process during the initial pressing stage, leading to a more significant hysteresis effect.

We extracted the measured pressure level of the three phantom tissue samples from the transient response at an applied displacement of 5 mm. The relationship between the sensor response and the estimated tissue elasticity plotted in Figure 7D shows a clear trend that phantom tissues with a higher stiffness exhibit a higher output pressure, which is consistent with the cyclic pressurizing results plotted in Figure 7A. This relationship can be fitted to the linear equation: Young’s modulus=0.05×P+0.29. This relationship allows for quantification of the mechanical properties of biotissue using the proposed sensor.

## 5. Demonstration of the Tactile Sensor on Surgical, Soft Robot

**Figure 8:**
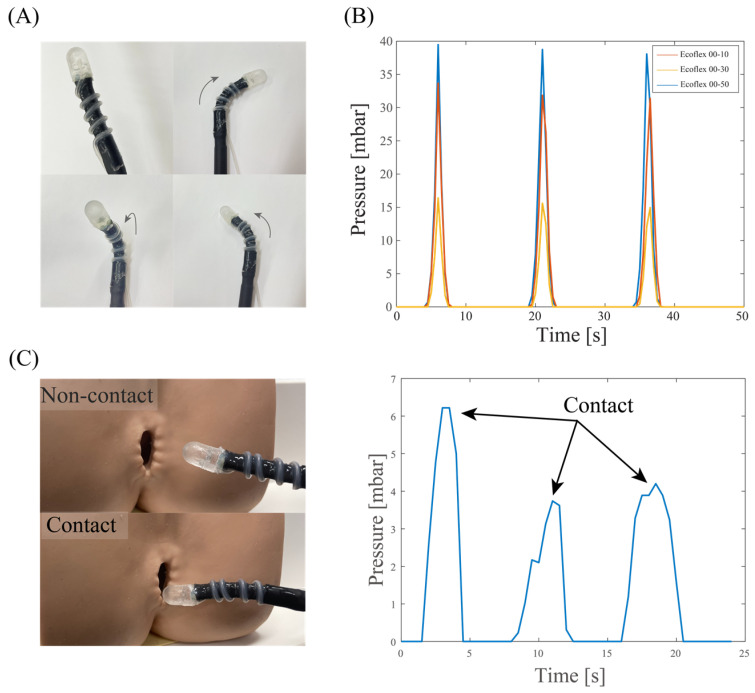
Demonstration of the as-fabricated sensor on soft robotic arm. (**A**) A photograph of the sensor integrated onto the robot. (**B**) Detection of tissues with different stiffness using the sensor-integrated soft robot. (**C**) Demonstration of the soft robot on a 3D organ model.

Figure 8A shows a photograph of a sensor-integrated soft medical robot, where the elastic air chamber is mounted on the top surface of the robot’s end effector. The fluidic configuration (air) of the tactile sensor enables seamless integration with the hydraulic soft robotics (liquid), where there is no direct electrical wiring posed to the inner tissue. The fabrication process and characteristics of our robotics system can be found in [35]. The elasticity of the silicone tube retains the mechanical compliance of the system, allowing the robot to generate different actuations such as bending in left, right, and out-of-plane directions, as shown in Figure 8A. This high degree of freedom in the robot’s motions is achieved by utilizing a set of three hydraulic tubes that can be independently or simultaneously actuated.

We demonstrated the capability of the soft robot in detecting tissues with different stiffness. Simultaneously actuating the three hydraulic tubes generates linear motion in the soft robot, which inserts a normal force to the targeted tissue. Figure 8B shows the sensor’s response when applying a constant displacement of 5 mm for 5 s to three types of phantom tissues. The sensor shows a higher spike output when interacting with the Ecoflex 00-50 sample, while exhibiting smaller outputs when pressing the Ecoflex 00-30 and 00-10 samples. The results clearly demonstrate the feasibility of using our device for in situ mechanical characterization and feedback control. Another demonstration on a 3D model of an anus clearly shows the touching events between the robot end effector and the tissue, suggesting the possibly for palpation applications through the colon tract, Figure 8C.

**Figure 9:**
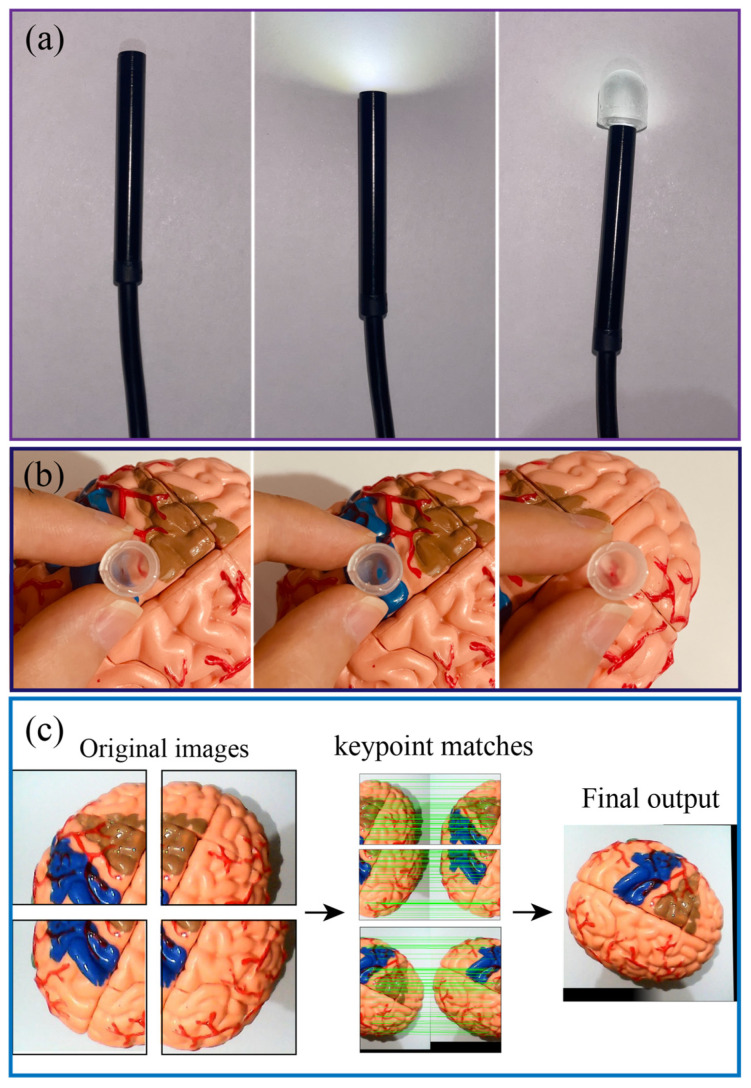
Demonstration of optical transparency of the tactile sensor. (**a**) LED light can pass through the PDMS chamber. (**b**) Texture of organ can be observed through the sensor. (**c**) Image processing to build a large field-of-view image.

Owing to the optical transparency of PDMS, our system can retain the on-site imaging assessment capability in conventional endoscopy. Figure 9a presents the optical transparency of the sensors when connect with an USB endoscope and a camera, clearly showing that the LED light can pass through the PDMS air chamber. Although the light intensity is slightly decreased when passing through the PDMS layer, this can be further improved by smoothing the PDMS surface using a high-resolution 3D-printed mould. The quality of the figure can be further improved when the sensor closely approaches the targeted tissues or organs, as shown in Figure 9b. It is noteworthy to mention that the field-of-view of the system could be small for each single imaging capture. However, a spatial imaging resolution of an organ can be obtained by scanning the robot in different locations and using the image stitching function (e.g., OpenCV’s Stitcher class) as demonstrated in Figure 9c. Overlapping the real-time processed image (enabled through the optical path of the sensor and optical fiber) with mechanical characterization data from the tactile sensor would provide meaningful information for palpation analysis.

## 6. Conclusions

This work develops a novel tactile sensor with mechanical flexibility and optical transparency for medical robotic control and palpation applications. The use of the PDMS air chamber for the pneumatic tactile sensors combined with soft microtubule artificial muscles (SMAM) offers excellent mechanical flexibility in surgical robotic systems. The tactile sensor exhibits excellent optical transparency, thanks to the transparency of PDMS, enabling the endoscope to capture high-quality visual information. The sensor was developed using a low cost and simple technique with a 3D-printed mould and PDMS casting to obtain a tactile sensor with a diameter of 10 mm and a height of 12 mm. Experimental data validate the use of this sensor for measuring the contact between the robotic end-effector with biotissue together with differentiating tissue stiffness ranging from 0 to 2.5 MPa, and the tactile sensor exhibits good repeatability and accuracy, suggesting a promising possibility to detect tumors through mechanical touch. We also successfully integrated the as-fabricated sensor with a soft medical robotics arm driven by hydraulic soft microtubule artificial muscles. The system exhibits excellent mechanical flexibility and a high degree of freedom, along with mechanical sensing and optical visualizing capability. These features demonstrate the potential for the development of an all-in-one surgical soft robotic system that can simultaneously perform on-site diagnosis and therapy.

## Figures and Tables

**Table 1:** Comparative of the recent high-performance sensors with different working mechanisms.

Reference	Working Mechanism	Range	Sensitivity	Structure	Material
[22]	Piezoresistive-based	0–10 KPa	0.572 kPa^−1^	Foam	GO-AgNF-PI sponge
[23]	Piezo-capacitive-based	0–10 KPa	1.2 kPa^−1^	Lotus mould substrate/AgNWs electrode	Ag NWs/PDMS/CPI
[24]	Piezoelectric-based	1–30 kPa	0.33 V kPa^−1^	Composite microfiber	P(VDF-TrFE)/BaTiO_3_
[25]	Triboelectric-based	1–10 N	N/A	PET fabric coat black phosphorus and particles	HCOENPs/BP/PET
[26]	Optical-based	0–1000 N	N/A	Composite disk	CaZnOS: Nd^3+^/epoxy

**Table 2:** Comprehensive performance comparison of tactile sensors.

Reference	Dimension	Measuring Range	Mechanical Flexibility (Drive)	Optical Transparency	Others
[28]	Diameter = 2 mm	0–0.3 N	Steerable robotic probe	No	Maximum hysteresis = 5.58%
[29]	Bulky and not suitable for in vivo	0–155 KPa	N/A	Blur	N/A
[30]		00-40 Shore hardness	N/A	Blur	Accuracy, sensitivity, and reliability > 90%
Proposed sensor	Diameter = 10 mm	0–2.5 MPa	SMAM	Yes	Sensitivity = 1.25 mbar

## Data Availability

Data available on request from the authors.
